# Case of Remarkable Contraction of the Stomach, near Its Centre

**Published:** 1816-01

**Authors:** Shirley Palmer


### *Case of remarkable Contraction of the Stomach, near its Centre.* By Shirley Palmer, M. D.

A Lady, of delicate and pleasing person, acute sensibility, and remarkably mild and engaging manners, became affected, about the age of puberty, with bronchocele. For the removal of this, the sponge-medicine was long and successfully administered. The swelling disappeared. From that period, however, the constitution became sensibly deranged. Indigestion, pain of the stomach with occasional vomiting, variable appetite, disordered bowels, and gradually-increasing weakness and emaciation, stood most conspicuous in the train of symptoms. All that parental tenderness could devise, all that medical science and attention could accomplish, was opposed in vain to the slow but steady ravages of the gastric malady. For although frequently and conspicuously retarded by the operation of medicinal remedies, or by excursions to the watering places or the coast, the progress of the complaint had never been permanently arrested. The friends of the lady attributed the origin of her sufferings to the employment of the burnt sponge. In this inference they were probably mistaken.

About the commencement of the year 1812, I had first an opportunity of observing this interesting case; and received from the patient herself the statement which I have just delivered. She was then in her thirty-seventh year, and unmarried.

Her emaciation was at that time extreme; her complexion fair and florid; her countenance commonly animated. Except when depressed by extreme pain or sickness, she possessed a considerable flow of spirits, and her conversation was sprightly. She referred the whole of her suffering to the epigastric region. Some degree of pain almost invariably came on in a short time after taking food; and, when violent, was rarely appeased until the contents of the stomach were discharged by vomiting. Reclination on the right side would at any time serve to bring on a paroxysm of pain and sickness, or to aggravate it when already excited. The contrary position, on the other hand, was always productive of relief, and hence constantly observed.* There was sometimes a short dry cough; frequently an acute pain under the left false ribs affecting respiration. The appetite for food was rarely impaired; in general, very keen. The action of the bowels was irregular, often torpid; their secretions variable. A state of sensible perspiration could never be induced. The functions of the pelvic organs were duly performed.

Defective nutrition was every where marked in striking characters; but the depression of muscular strength was not so great as appearances would seem to indicate. There was no œdema of the extremities,—no induration, nor tension, and but slight soreness on pressure, in the epigastric region. The tongue commonly presented a clear and moist surface. The arterial pulsations at the wrist were weak, regular, usually about eighty in the minute.

The paroxysms of pain and sickness recurred at uncertain intervals, and varied considerably in duration and vehemence. Sometimes they were truly terrible, and moreover aggravated by distressing palpitations and disposition to syncope. They commonly terminated in rejection of

---

* This circumstance is clearly elucidated by the dissection. Whenever our patient lay on the right side, the whole contents of the cardiac portion of the stomach must necessarily press on that morbid part of the organ which formed the seat of the stricture; and unable to pass, on account of the smallness of the aperture, produced pain, irritation, and vomiting. We also may hence understand why aliment, imperfectly masticated, always exerted on the stomach an influence peculiarly prejudicial. Cautious contemplation of the effects resulting from particular positions of a patient's body may, perhaps, occasionally throw some light upon the diagnosis in organic lesions of the stomach.

the whole contents of the stomach. The matter vomited had an extremely acid taste and smell; now and then it exhibited a pus like appearance. Thrice I observed it to be slightly streaked with blood. During the more violent attacks, the pain extended from the epigastrium to the spine, the thorax, and umbilical region; and the appearances of languor, exhaustion, and distress were often such as to impress the observer with an idea that she could never again raise her head from the pillow on which it was supported. Yet the rapidity with which, after a paroxysm was gone by, the powers of exertion and the appetite were restored, could scarcely be contemplated without astonishment. Opium and hot water, administered internally; hot fomentations to the epigastric region; lying down on the left side, or inclination of the body forwards, generally mitigated the severity of the pain or shortened its duration. The fæces, voided on the decline of a paroxysm, were fœtid, unnatural, and occasionally tinged with blood; the urinary secretion high coloured, depositing a copious sediment. The respite which succeeded was often perfect; sometimes, under favourable circumstances, of several days' continuance. Exertions of mind or body, carried so far as to induce fatigue; sudden atmospheric vicissitudes; and loss of rest, seemed always to operate in predisposing the system to a repetition of the paroxysm: yet, little observation was requisite to convince me that food, of an indigestible nature, prone to fermentation, or imperfectly masticated, might be regarded as the principal and direct agent in its re-production. Hence the propriety of a rigorous dietetic plan, and of such remedies as would most effectually counteract the fermenting process in the stomach, suggested itself. The paroxysms, I should observe, were always accompanied or preceded by a sense of heat and distension in the gastric organ.

The diet was directed to consist of plain, mild and nutritious aliment. All vinous and distilled liquids, every substance prone to fermentation, were strictly prohibited. The necessity of small but frequent ingestion, and of cautious mastication of the food, was strongly insisted on. Lime-water was prescribed for common drink; a combination of pure potass, with narcotics, to be administered at proper intervals. Great attention to regulate the functions of the intestinal tube was also employed; but here unusual difficulty was encountered. The milder aperients failed altogether of effect. Drastic purgatives, while, by the severity of their operation, they induced great pain, exhaustion, and distress, served only to render the action of the bowels subsequently more torpid. Preparations of quicksilver, aloes, sulphate of magnesia, and many other medicinal articles, were tried in vain. A simple combination was at length found adequate to an object which more potent remedies had failed for many months to accomplish. Rhubarb powder and magnesia, exhibited every night in a light infusion of ginger, procured daily one or two natural evacuations from the bowels; and to this circumstance the subsequently ameliorated condition of our patient may, perhaps in great measure, be attributed.* The lime-water was taken in very considerable quantities, particularly on the accession of pain, acidity, or nausea of the stomach. These symptoms it very often relieved; and thus many an impending paroxysm was averted.

The year 1812 passed over with a sensible amendment in the situation of our patient. For neither did the paroxysms of pain and vomiting recur so frequently, nor were they in their severity, duration, or exhausting consequences, so terrible as heretofore. Commonly too their reproduction might be traced to some deviation from the plan of diet, some moral cause or corporeal exertion, which, operating on the morbid stomach, still farther deranged that organ, already almost inadequate to the due performance of its functions, and exquisitely susceptible to the influence of every moral and external agent. Towards the close of this year, the pulse evinced, upon some occasions, a slight irregularity; and the palpitations of the heart, frequently attendant on the paroxysms of gastric disturbance, assumed a somewhat aggravated character.

Appearances, in the commencement of 1813, were still more auspicious. The regularity of the alvine discharges was now fully established. The attacks of pain and vomiting had signally decreased both in frequency and violence. An unusual expression of vigour and animation was marked upon the countenance. The muscular forces seemed rising from their long depression. These changes, favourable as they were, led to unfortunate consequences. Exemption from severe suffering at length inspired a delusive sense of security. The restrictions with

---

* In many cases of obstinate and habitual constipation, I have found this simple combination, administered regularly, succeed in restoring the bowels to their natural state, when every other article of the materia medica, in various doses and combinations, had previously been tried in vain.

respect to diet and exercise began to be less rigidly observed. On Tuesday, May 11th, our patient, after taking some alimentary substance of a highly indigestible nature, walked out to the distance of a mile; an exertion of which, for many months, she had been incapable. The greater part of her way led up a steep ascent. An unclouded sun had not long passed the meridian. Great faintness and exhaustion were the immediate consequences. Violent pain of the stomach, vomiting, rigors, soreness of the abdomen, thirst, dry skin, accelerated circulation, came on in rapid succession. She was conveyed home and put to bed. Obstinate rejection of food and medicine, and complete inaction of the bowels, were added to the train of symptoms. In fact, on the following day all the phænomena of intestinal inflammation were developed in deepest characters. For the first three days, every effort to allay the vomiting and remove the constipation failed. At length, on Saturday the 15th, it was removed; but the system was too much exhausted to rally. Our patient sank on the afternoon of the ensuing day.

The body was examined by myself on Wednesday the 19th, in the presence of several professional gentlemen.

*External Appearances.* Emaciation extreme — no œdematous swelling: abdomen tumid and discoloured.—*Internal appearances.* On opening the abdomen, our attention was forcibly arrested by the extraordinary size and figure of the stomach. It lay obliquely across the abdominal cavity, which it almost wholly occupied, from the left hypochondrium to the hollow of the right ilium. Near the middle of the organ was observed a contraction, as deep and strongly marked as though a thin ligature had been passed tightly around it; and thus presenting the appearance of two distinct stomachs. Nothing unnatural displayed itself in the intimate structure of the peritonæal covering. On opening the cardiac division of the organ, great difficulty was found in passing the little finger through the narrow ring which separated it from the pyloric portion. This aperture of communication did not suffer itself to be dilated: the internal membrane, in the whole of the circle, was perfectly smooth, and of a natural colour; but all the coats of the stomach seemed thickened at the strictured part, and for some distance around, as though by deposition of lymph in the cellular membrane connecting them. The internal coat, however, presented, in the neighbourhood of the contraction, three separate and nearly circular patches of a deepish pink blush. Two of them were situated in the cardiac, one in the pyloric division of the stomach. They differed in size, and had a regularly defined margin. On very close examination, this appearance was seen to arise from clusters of small, numerous, and thickly disposed papillæ, like minute granulations of the middle coat protruding through correspondent orifices of the internal. This morbid structure was only visible in a strong light, and became exceedingly faint and indistinct after a few hours' maceration in water. The orifice of the pylorus was unusually small; unusually strong the band of muscular fibres surrounding it. The condition of the internal coat was, in all other respects, natural. Traces of high inflammation were visible in the whole track of the duodenum, and in the commencement of the jejunum. One portion of the former intestine had an almost livid appearance, but was not disorganized. About half an inch below the pylorus, arising abruptly, and in a sinistro-lateral direction from the duodenum, was a curious pouch, just capable of receiving the first joint of a large thumb. The parietes of it precisely resembled in structure those of the tube from which it sprang. No obvious solution of continuity in the coats of the duodenum had taken place to form this pouch: it was nearly empty. The pancreas was somewhat more dense and firm in structure than common, but presented no other morbid characters. The liver, the spleen, and all the other abdominal and pelvic viscera, were in a natural condition. The aortic valves were unusually strong and rigid, verging towards a cartilaginous degeneration. The heart itself and remaining thoracic organs were perfectly sound.

---

It may be questioned by some of my readers, whether the contraction of the stomach, here described, were in reality the consequence of morbid action, or had existed in the rudiments of the fœtus, as a peculiarity of structure; whether, in fact, it should be deemed a lesion, or original malformation of the gastric organ. Morgagni has recorded three cases in which this contraction of the stomach was found upon dissection, although no symptom, developed during life, had indicated the existence of such a state. In none of these cases, however, does the contraction appear to have been extreme. Yet an example is recorded by Blasius, wherein the aperture between the two divisions of a strictured stomach was exceedingly strait; and the subject of the observation, in addition to good general health, possessed a voracious appetite. Notwithstanding this evidence, we are, I think, justified in concluding, that the stricture discovered in our patient had taken place subsequently to birth, and hence constituted a morbid state: first, because, for the greatest part of her youth, she had been quite healthy; and secondly, because, after death, no other lesion of the abdominal viscera was found, capable of explaining a terrible malady of more than twenty years' duration; the actual seat of which was moreover unequivocally marked by the external phænomena presented during life. It is, after all, very possible that the central contraction of the stomach may sometimes exist as a peculiarity of original conformation; many respectable writers,* besides Morgagni and Blasius, having recorded examples of it, where vomiting, one of the essential symptoms of *morbid* stricture, had never been experienced.

In the three circular patches, noted on the internal coat of the stomach, chronic inflammation had probably been long established. Had life been sufficiently protracted, would they not have become the seat of the ulcerative process? This question I should be disposed to answer in the affirmative. Ulceration, however, it should be observed, had no where actually taken place. Hence the pus-like appearance of the matter occasionally ejected was delusive. It could be nothing but a morbid secretion.

That the fatal event was determined, not by the lesion of the stomach, but by the intestinal inflammation, the history and dissection of the case alike incontestably prove. The influence of indigestible aliment and undue muscular exertion, in exciting the alvine tube to a destructive increase of action, neither can nor should be overlooked. The pouch, apparently formed by partial dilatation of the duodenal membranes, admits of no satisfactory explanation.

Three cases of central gastric contraction, evidently the source, if not originally the consequence, of morbid actions, may be found in the writings of Valsalva, Blasius, and Morgagni. For other examples of a similar lesion, I have searched in vain the works of Lieutaud, Portal, Baillie, Monro, Pemberton, Stone, and Chardel, and many of the British and continental Journals of medicine. The "*Handbuch der Pathologischen Anatomie,*" of Voigtel, I am not fortunate enough to possess.†

* Riolanus, Hester, Fantonus, and others.

† This is an excellent "Manual of Morbid Anatomy," in the German

I shall conclude this paper in our next Number, with an attempt to trace the diagnosis between central stricture of the stomach, and those other principal lesions of the abdomen, which in the leading characters it most closely resembles. The importance of correct discrimination is unquestionable, even in those forlorn maladies against which medicine can exert only the feeble arm of palliation, and wherein little is left for the physician but to deplore the impotency of his art.

*Tamworth, December 4th,* 1815.

language. No *analysis* of it has yet appeared in the English Journals. We shall endeavour to obtain it, for the purpose of a comprehensive review.

